# Prevalence and Antifungal Susceptibility of *Candida parapsilosis* Species Complex in Eastern China: A 15-Year Retrospective Study by ECIFIG

**DOI:** 10.3389/fmicb.2021.644000

**Published:** 2021-03-04

**Authors:** Jian Guo, Min Zhang, Dan Qiao, Hui Shen, Lili Wang, Dongjiang Wang, Li Li, Yun Liu, Huaiwei Lu, Chun Wang, Hui Ding, Shuping Zhou, Wanqing Zhou, Yingjue Wei, Haomin Zhang, Wei Xi, Yi Zheng, Yueling Wang, Rong Tang, Lingbing Zeng, Heping Xu, Wenjuan Wu

**Affiliations:** ^1^Department of Laboratory Medicine, Shanghai East Hospital, Tongji University School of Medicine, Shanghai, China; ^2^Department of Laboratory Medicine, Ruijin Hospital, Shanghai Jiao Tong University School of Medicine, Shanghai, China; ^3^Department of Laboratory Medicine, Changhai Hospital, Naval Medical University (Second Military Medical University), Shanghai, China; ^4^Department of Laboratory Medicine, The First Affiliated Hospital of USTC, Hefei, China; ^5^Department of Laboratory Medicine, Children’s Hospital, Shanghai Jiao Tong University School of Medicine, Shanghai, China; ^6^Department of Laboratory Medicine, Lishui Municipal Central Hospital, Lishui, China; ^7^Department of Laboratory Medicine, Jiangxi Provincial Children’s Hospital, Nanchang, China; ^8^Department of Laboratory Medicine, Nanjing Drum Tower Hospital, The Affiliated Hospital of Nanjing University Medical School, Nanjing University, Nanjing, China; ^9^Department of Laboratory Medicine, Renji Hospital, Shanghai Jiao Tong University School of Medicine, Shanghai, China; ^10^Department of Clinical Laboratory, The Second Affiliated Hospital of Soochow University, Suzhou, China; ^11^Department of Clinical Laboratory, Shandong Provincial Hospital Affiliated to Shandong First Medical University, Jinan, China; ^12^Department of Laboratory Medicine, Shanghai General Hospital, Shanghai Jiao Tong University School of Medicine, Shanghai, China; ^13^Department of Laboratory Medicine, The First Affiliated Hospital of Nanchang University, Nanchang, China; ^14^Department of Laboratory Medicine, The First Affiliated Hospital of Xiamen University, Xiamen, China

**Keywords:** antifungal susceptibility, *Candida parapsilosis*, *Candida metapsilosis*, *Candida orthopsilosis*, China

## Abstract

*Candida parapsilosis* complex is one of the most common non-*albicans Candida* species that cause candidemia, especially invasive candidiasis. The purpose of this study was to evaluate the antifungal susceptibilities of both colonized and invasive clinical *C. parapsilosis* complex isolates to 10 drugs: amphotericin (AMB), anidulafungin (AFG), caspofungin (CAS), micafungin (MFG), fluconazole (FLZ), voriconazole (VRZ), itraconazole (ITZ), posaconazole (POZ), 5-flucytosine (FCY), and isaconazole (ISA). In total, 884 *C. parapsilosis* species complex isolates were gathered between January 2005 and December 2020. *C. parapsilosis*, *Candida metapsilosis*, and *Candida orthopsilosis* accounted for 86.3, 8.1, and 5.5% of the cryptic species, respectively. The resistance/non-wild-type rate of bloodstream *C. parapsilosis* to the drugs was 3.5%, of *C. metapsilosis* to AFG and CAS was 7.7%, and of *C. orthopsilosis* to FLZ and VRZ was 15% and to CAS, MFG, and POZ was 5%. The geometric mean (GM) minimum inhibitory concentrations (MICs) of non-bloodstream *C. parapsilosis* for CAS (0.555 mg/L), MFG (0.853 mg/L), FLZ (0.816 mg/L), VRZ (0.017 mg/L), ITZ (0.076 mg/L), and POZ (0.042 mg/L) were significantly higher than those of bloodstream *C. parapsilosis*, for which the GM MICs were 0.464, 0.745, 0.704, 0.015, 0.061, and 0.033 mg/L, respectively (*P* < 0.05). The MIC distribution of the bloodstream *C. parapsilosis* strains collected from 2019 to 2020 for VRZ, POZ, and ITZ were 0.018, 0.040, and 0.073 mg/L, significantly higher than those from 2005 to 2018, which were 0.013, 0.028, and 0.052 mg/L (*P* < 0.05). Additionally, MIC distributions of *C. parapsilosis* with FLZ and the distributions of *C. orthopsilosis* with ITZ and POZ might be higher than those in Clinical and Laboratory Standards Institute studies. Furthermore, a total of 143 *C. parapsilosis* complex isolates showed great susceptibility to ISA. Overall, antifungal treatment of the non-bloodstream *C. parapsilosis* complex isolates should be managed and improved. The clinicians are suggested to pay more attention on azoles usage for the *C. parapsilosis* complex isolates. In addition, establishing the epidemiological cutoff values (ECVs) for azoles used in Eastern China may offer better guidance for clinical treatments. Although ISA acts on the same target as other azoles, it may be used as an alternative therapy for cases caused by FLZ- or VRZ-resistant *C. parapsilosis* complex strains.

## Introduction

*Candida albicans* and emerging non-*albicans Candida* species can result in superficial infections of the oral and vaginal mucosa, as well as invasive candidiasis, such as bloodstream infections and deep-tissue infections. These invasive infections are associated with high mortality of about 70% (Pappas et al., 2018). *C. albicans* is the most common and aggressive species causing *Candida* infections around the world. However, over the past few decades, non-*albicans Candida* species such as *Candida glabrata*, *Candida parapsilosis*, and *Candida tropicalis* have also become health concern (Chow et al., 2008; Silva et al., 2012; Guinea, 2014; Guo et al., 2017). Among them, *C. parapsilosis* is well known for its threat to the patients undergoing invasive medical interventions, as it is considered to be one of the leading causes of catheter-related infections and can form enhanced biofilms on central venous catheters (CVCs) and other medical implants (Trofa et al., 2008; Pfaller et al., 2014; Strollo et al., 2016; Tóth et al., 2019). *C. parapsilosis* is also the second or third most commonly isolated *Candida* species in the intensive care units (ICUs) (Magobo et al., 2017; Asadzadeh et al., 2019). In contrast to *C. albicans*, horizontal transmission is another characteristic of *C. parapsilosis*, which allows the species to spread through contaminated medical equipment and medical staff in the clinic, leading to crossover infections between patients (García San Miguel et al., 2004; Kuhn et al., 2004; Vaz et al., 2011).

*Candida parapsilosis*, *C. metapsilosis*, and *C. orthopsilosis* are three species of the *C. parapsilosis* species complex (Tavanti et al., 2005). The prevalence of *C. parapsilosis* is the highest among the cryptic species. A 6-year multicenter study in Iran reported that the proportions of *C. orthopsilosis* and *C. metapsilosis* were quite small, comprising 5.3 and 0.17% of all *C. parapsilosis* species complex isolates (Arastehfar et al., 2019). While in Argentina and India, *C. orthopsilosis* may account for a higher proportion, reaching 40% of the cryptic species (Maria et al., 2018; Vigezzi et al., 2019). *C. parapsilosis* complex isolates were found susceptible to most of the antifungal agents (Modiri et al., 2019; Vigezzi et al., 2019). The multicenter studies in China by the China Hospital Invasive Fungal Surveillance Net (CHIF-NET) performed in 2015, 2018, and 2020 all reported low resistance/non-wild-type (NWT) rate of *C. parapsilosis* complex isolates to azoles (<6%) (Xiao et al., 2015, 2018, 2020). The multicenter study in Iran also reported four NWT *C. orthopsilosis* isolates for itraconazole (ITZ) (Arastehfar et al., 2019). Yet, the work in India reported high resistance to fluconazole (FLZ) of *C. parapsilosis* (Maria et al., 2018). However, all of the studies lasted only 2–6 years, lacking long-term studies.

Additionally, the difference in antifungal susceptibilities between the three cryptic species were described. Vigezzi et al. (2019) reported lower minimal inhibitory concentrations (MICs) with *C. parapsilosis* than *C. orthopsilosis* for ITZ and higher MIC values for echinocandins (*P* < 0.01). Similarly, Gil-Alonso et al. (2019) reported that *C. metapsilosis* was the most susceptible species to echinocandins, followed by *C. orthopsilosis* and *C. parapsilosis*. However, the number of strains used in these studies were quite limited so that these results may not able to uncover the difference clearly.

Furthermore, the Clinical and Laboratory Standards Institute (CLSI) updated its document M60-Ed2 in June 2020, illustrating that the clinical breakpoints can only be used for *C. parapsilosis*; otherwise, the epidemiological cutoff values (ECVs) recommended by M59-Ed3 should be applied for *C. metapsilosis* and *C. orthopsilosis.* No large-scale study, except the programs associated with CLSI, has been performed since then.

Notably, only the *C. parapsilosis* complex isolates from invasive candidiasis have been studied for their antifungal susceptibilities so far. *C. parapsilosis* complex are opportunistic pathogens that may transition from colonization to invasion; therefore, we believe that it is also worth studying colonized isolates. We launched the Eastern China Invasive Fungi Infection Group (ECIFIG), a multicenter institute, in 2019 in Shanghai to supervise the *Candida* strains isolated from both colonization and invasion sites and to improve rapid fungal diagnosis, therapeutic drug monitoring, and clinical intervention teams.

Therefore, this 15-year multicenter study collected a total of 884 colonized and invasive *C. parapsilosis* complex isolates in eastern China, with the aim of investigating their epidemiological characteristics and antifungal susceptibility distributions systematically with nine common antifungal drugs as well as isavuconazole (ISA), a drug whose ECV was first reported in CLSI M59-Ed3 for *C. duobushaemulonii* only, applying the clinical breakpoints and ECVs updated in CLSI M60-Ed2 and M59-Ed3.

## Materials and Methods

### Strains

For this retrospective study, a total of 884 *C. parapsilosis* complex clinical isolates (763 *C. parapsilosis*, 49 *C. orthopsilosis*, and 72 *C. metapsilosis*) were collected from 835 patients with more than one episode of candidiasis. The isolates were gathered from different tertiary hospitals of ECIFIG between 2005 and 2020. Among the seven provinces in Eastern China, the majority of the isolates were from Shanghai (*n* = 520), Jiangxi (*n* = 240), and Fujian (*n* = 64) (Table 1). For each isolate, the collected information included age and gender of the patient, date of sample collection, specimen type, body site of isolation, and the ward location of the patient at the time of sample collection. Strains were isolated from clinical samples of patients with bloodstream and non-bloodstream fungal infections.

**TABLE 1:** Number of *C. parapsilosis* complex isolates collected from different hospitals.

Hospitals		No. of isolates (%)		
		
		*C. parapsilosis* (*n* = 763)	*C. metapsilosis* (*n* = 72)	*C. orthopsilosis* (*n* = 49)
Shanghai	East Hospital	185 (24.2)	2 (2.8)	4 (8.2)
	Ruijin Hospital	35 (4.6)		6 (12.2)
	Shanghai Children’s Hospital	34 (4.5)	1 (1.4)	1 (2)
	Changhai Hospital	26 (3.4)	1 (1.4)	11 (22.4)
	Changzheng Hospital	26 (3.4)		
	Shanghai General Hospital	26 (3.4)	1 (1.4)	
	Shanghai Tenth People’s Hospital	23 (3)	3 (4.2)	
	Eye and Ent Hospital of Fudan University	19 (2.5)	5 (6.9)	2 (4.1)
	East Brach of Renji Hospital	14 (1.8)	2 (2.8)	1 (2)
	Shanghai Public Health Clinical Center	13 (1.7)		
	West Brach of Renji Hospital	12 (1.6)	3 (4.2)	
	Zhongshan Hospital	11 (1.4)		
	Children’s Hospital of Fudan University	9 (1.2)		1 (2)
	Huadong Hospital	9 (1.2)		
	Shanghai Children’s Medical Center	8 (1)		
	South Brach of Renji Hospital	6 (0.8)		
	Huashan Hospital	5 (0.7)		
	Shanghai Eastern Hepatobiliary Surgery Hospital	4 (0.5)		
	Shanghai Chest Hospital	4 (0.5)		
	Shanghai Cancer Center	4 (0.5)		
	Shanghai Pudong New Area Gongli Hospital	3 (0.4)		
	Total	476 (62.4)	18 (25)	26 (53.1)
Jiangxi	The First Affiliated Hospital of Nanchang University	168 (22)	41 (56.9)	21 (42.9)
	Jiangxi Provincial People’s Hospital	9 (1.2)		
	Jiangxi Provincial Children’s Hospital	1 (0.1)		
	Total	178 (23.3)		
Fujian	The First Affiliated Hospital of Xiamen University	51 (6.7)	11 (15.3)	2 (4.1)
Zhejiang	Zhejiang Lishui Central Hospital	19 (2.5)		
Anhui	Anhui Provincial Hospital	18 (2.4)	2 (2.8)	
Jiangsu	The First Affiliated Hospital of Soochow University	9 (1.2)		
	Nanjing Drum Tower Hospital	8 (1)		
	Total	17 (2.2)		
Shandong	Shandong Provincial Hospital	4 (0.5)		

### Strains Identification

All isolates were first identified by biochemical methods and then confirmed by matrix-assisted laser desorption ionization–time of flight mass spectrometry (MALDI-TOF) (Autof ms1000, Autobio). Sequencing of the internal transcribed spacer (ITS) ribosomal DNA (rDNA) (ITS1/ITS4) region was performed for definitive species identification.

### Criteria for Study Inclusion

We collected all *C. parapsilosis* complex isolates recovered from the blood and non-blood of patients and included them in this study. Isolates from bronchoalveolar lavage fluid (BALF), CVC tips, and the gastrointestinal tracts of patients with invasive infections were tested. Isolates from sputum, urine, genital tract, and other places considered to be colonizers were also collected as non-blood strains. Isolates of the same species with the same susceptibility or resistance biotype profile from the same site of the same patient at different times were considered duplicates and were excluded.

### Criteria for Grouping

Since ECIFIG was launched in 2019 and started to collect all the strains isolated clinically, we divide the bloodstream *C. parapsilosis* isolates into two groups—2005–2018 and 2019–2020—to study the difference for MIC distributions between the two groups.

### Antifungal Susceptibility Testing

The *in vitro* susceptibility of the 741 *C. parapsilosis* complex strains to nine antifungal drugs, amphotericin B (AMB, 0.12–8 mg/L), anidulafungin (AFG, 0.015–8 mg/L), caspofungin (CAS, 0.008–8 mg/L), micafungin (MFG, 0.008–8 mg/L), FLZ (0.12–256 mg/L), VRZ (0.008–8 mg/L), ITZ (0.015–16 mg/L), posaconazole (POZ, 0.008–8 mg/L), and 5-flucytosine (FCY, 0.06–64 mg/L) were determined by the Sensititre YeastOne^TM^ YO10 methodology (Thermo Fisher Scientific, Waltham, MA, United States) following the manufacturer’s instructions. The *in vitro* antifungal susceptibility tests of the other 143 *C. parapsilosis* complex strains collected in 2020 were conducted using the Sensititre YeastOne^TM^ CMC1JHY methodology (Thermo Fisher Scientific), which is a new plate for research only, in which AMB is replaced with isavuconazole (ISA, 0.015–16 mg/L), and the concentration of CAS is changed from 0.008–8 mg/L to 0.06–4 mg/L. For these 143 strains, the *in vitro* susceptibilities to AMB were determined by the AMB microbroth dilution kit (BIO-KONT^®^, Wenzhou, China), which is also for scientific research only, with the drug concentrations ranging from 0.125 to 8 mg/L. *C. parapsilosis* (ATCC 22019) and *C. krusei* (ATCC 6258) standard strains were used as quality controls. After being incubated at 35°C for 24 h, the MIC endpoints were determined following the manufacturer’s instructions. For Sensititre YeastOne^TM^ YO10 and CMC1JHY (Thermo Fisher Scientific), MIC was defined as the lowest drug concentration at which the color in the well changed from red to blue. For the AMB microbroth dilution kit (BIO-KONT^®^), MIC was defined as the lowest concentration at which there was 100% growth inhibition. MIC_50_ and MIC_90_ were defined as the MICs required to inhibit the growth of 50 and 90% of the organisms, respectively. Data were interpreted based on the clinical breakpoints recommended by the CLSI M60-Ed2. Accordingly, the ECVs recommended by CLSI M59-Ed3 were applied if further species determination identified one of the cryptic species within the complex.

### Ethical Approval

This retrospective study was approved by the ethics committee of Shanghai East Hospital, Tongji University School of Medicine. The need for informed consents was waived by the Clinical Research Ethics Committee [(2017) Pre-examination No. 026].

### Statistical Analysis

The results were initially evaluated to assess if the data exhibited asymmetry and/or high variances using the Mann–Whitney test to compare the MIC distributions between non-bloodstream and bloodstream isolates as well as to compare the MIC distributions of bloodstream *C. parapsilosis* isolates between 2015–2018 and 2019–2020. The Kruskal–Wallis tests were used to compare the MIC distributions between the three cryptic species. *P* < 0.05 indicated statistical significance. Statistical analyses were performed using IBM SPSS for Windows v22.0 (IBM, Armonk, NY, United States). Antifungal susceptibility results that were “≤ the lowest concentration” were defaulted as “= the lowest concentration” for statistical analysis. Figures were made using GraphPad Prism v8.0 (GraphPad Software, Inc., San Diego, CA, United States).

## Results

### Epidemiological Characteristics

The number of *C. parapsilosis* strains isolated were 334 (43.8%) from 2019 to 2020 and 429 (56.2%) from 2005 to 2018; *C. metapsilosis* 30 (41.7%) from 2019 to 2020 and 42 (58.3%) from 2005 to 2018; and *C. orthopsilosis* 15 (30.6%) from 2019 to 2020 and 34 (69.4%) from 2005 to 2018 (Table 2). Regarding patients with isolated strains, the age distributions among the three species were similar: 54.46 ± 23.29 years for *C. parapsilosis*, 52.38 ± 19 years for *C. metapsilosis*, and 51.21 ± 23.98 years for *C. orthopsilosis*. In addition, strains were nearly two times more frequently isolated from male than female patients. The variety of sample sources of *C. parapsilosis* was higher than that of *C. orthopsilosis* and *C. metapsilosis*. *C. parapsilosis* strains were mainly isolated from blood (37.1%), followed by sputum (11.5%), urine (10.4%), stool (7.2%), and CVC (6.6%). The proportion of *C. orthopsilosis* strains isolated from blood was highest (40.8%), followed by stool (14.3%), vagina (10.2%), sputum (8.2%), urine (8.2%), and CVC (4.1%). The percentage of strains isolated from blood was the lowest for *C. metapsilosis* among the three species (18.1%), followed by sputum (18.1%), stool (15.3%), and vagina (11.1%). The proportion of *C. metapsilosis* isolated from CVC was also the lowest among the three species (2.8%). The top 10 ward where *C. parapsilosis* complexes were isolated were ICU (12.4%), Oncology (11.5%), Emergency Medicine (8.9%), Gynecology (7.8%), Gastroenterology (7.0%), Respiratory Medicine (6.9%), Nephrology (5.9%), Hepatobiliary and Pancreatic Surgery (5.3%), Neonatology (4.0%), and Otorhinolaryngology (3.6%).

**TABLE 2:** Epidemiological characteristics of the *C. parapsilosis* complex isolates.

Characteristics		No. of isolates (%)		
		
		*C. parapsilosis* (*n* = 763)	*C. metapsilosis* (*n* = 72)	*C. orthopsilosis* (*n* = 49)
Year	2005–2018	429 (56.2)	42 (58.3)	34 (69.4)
	2019–2020	334 (43.8)	30 (41.7)	15 (30.6)
Gender	Male	491 (64.4)	49 (68.1)	31 (63.3)
	Female	272 (35.6)	23 (31.9)	18 (36.7)
Source	Blood	283 (37.1)	13 (18.1)	20 (40.8)
	Sputum	88 (11.5)	13 (18.1)	4 (8.2)
	Urine	79 (10.4)	6 (8.3)	4 (8.2)
	Stool	55 (7.2)	11 (15.3)	7 (14.3)
	Central venous catheter	50 (6.6)	2 (2.8)	2 (4.1)
	Secretions	33 (4.3)		2 (4.1)
	Ear	21 (2.8)	6 (8.3)	2 (4.1)
	Pus	20 (2.6)	2 (2.8)	
	Ascites	19 (2.5)	1 (1.4)	
	Bile	18 (2.4)		
	Vagina	15 (2)	8 (11.1)	5 (10.2)
	Drainage	15 (2)	3 (4.2)	
	Pleural fluid	11 (1.4)	1 (1.4)	
	Tissue	11 (1.4)	1 (1.4)	1 (2)
	Peritoneal dialysis fluid	8 (1)		1 (2)
	Cerebrospinal fluid	8 (1)		
	Wound	6 (0.8)		
	Bronchoalveolar lavage fluid	4 (0.5)		
	Puncture fluid	4 (0.5)		
	Synovial fluid	4 (0.5)	1 (1.4)	
	Puncture needle	3 (0.4)		
	Nail	3 (0.4)	3 (4.2)	1 (2)
	Dialyzate	2 (0.3)		
	Donor kidney lavage fluid	1 (0.1)	1 (1.4)	
	Gastric juice	1 (0.1)		
	Suction tube	1 (0.1)		

### Antifungal Susceptibility Results

#### Resistance/NWT Rates of the *Candida parapsilosis* Complex Isolates

Among the 283 bloodstream *C. parapsilosis*, there were 3.2% SDD and 3.5% R to FLZ, 1.4% I and 2.5% R to VRZ, 0.4% I to MFG, 0.4% NWT to ITZ, 1.4% NWT to POZ, and 1.1% NWT to AMB (Supplementary Table 2). Among the 13 bloodstream *C. metapsilosis*, there were 7.7% NWT to AFG and 7.7% NWT to CAS. Among the 20 bloodstream *C. orthopsilosis*, there were 5% NWT to CAS, 5% NWT to MFG, 15% NWT to FLZ, 15% NWT to VRZ, and 5% NWT to POZ.

Among the 480 non-bloodstream *C. parapsilosis*, there were 4.4% SDD and 3.1% R to FLZ, 1.3% I and 1.5% R to VRZ, 0.4% I and 0.2% R to CAS, 1.5% I to AFG, 0.2% NWT to ITZ, 0.6% NWT to POZ, and 7.3% NWT to AMB. Among the 59 non-bloodstream *C. metapsilosis*, there were 10.2% NWT to AFG, 20.3% NWT to CAS, 3.4% NWT to VRZ, and 1.7% NWT to AMB. Among the 29 non-bloodstream *C. orthopsilosis*, there were 3.4% NWT to CAS, 3.4% NWT to MFG, 10.3% NWT to FLZ, 6.9% NWT to VRZ, and 3.4% NWT to POZ.

### MIC Values of the Nine Drugs Compared by Source (Non-bloodstream vs. Bloodstream)

**FIGURE 1:**
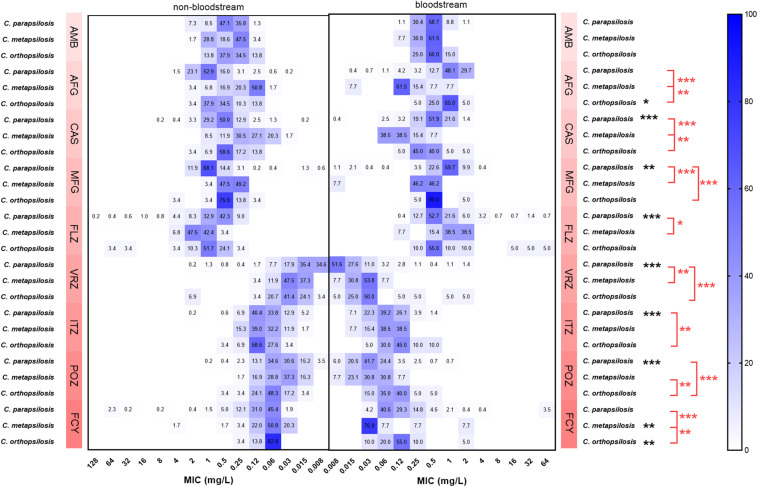
Comparison of the proportions of minimum inhibitory concentration (MIC) distributions of *C. parapsilosis* complex isolates between bloodstream and non-bloodstream samples. Black *indicates 0.01 < *P* < 0.5, statistically significant differences of MIC values between bloodstream and non-bloodstream isolates; black **indicates 0.001 < *P* < 0.01, black ***indicates *P* < 0.001. Red *indicates 0.01 < *P* < 0.5, statistically significant differences of MIC values between bloodstream *C. parapsilosis* complex species; red **indicates 0.001 < *P* < 0.01, red ***indicates *P* < 0.001. FLZ, fluconazole; VRZ, voriconazole; ITZ, itraconazole; POZ, posaconazole; MFG, micafungin; AFG, anidulafungin; CAS, caspofungin; AMB, amphotericin B; FCY, 5-flucytosine.

Although the general trend of the MIC ranges of the nine drugs for *C. parapsilosis*, *C. metapsilosis*, and *C. orthopsilosis* isolates were similar between bloodstream and non-bloodstream sources (Figure 1 and Supplementary Table 1), for some drugs, their MIC values differed between the two sources. The GM MICs of non-bloodstream *C. parapsilosis* for CAS, MFG, FLZ, VRZ, ITZ, and POZ were 0.555, 0.853, 0.816, 0.017, 0.076, and 0.042 mg/L, respectively. These GM MICs were significantly higher than those of bloodstream *C. parapsilosis*, for which the GM MICs were 0.464, 0.745, 0.704, 0.015, 0.061, and 0.033 mg/L, respectively, for CAS, MFG, FLZ, VRZ, ITZ, and POZ (*P* < 0.05). The GM MIC of non-bloodstream *C. metapsilosis* for FCY (0.071 mg/L) was significantly higher than that of bloodstream isolates (0.051 mg/L) (*P* < 0.05). In contrast, the GM MICs of non-bloodstream *C. orthopsilosis* for AFG and FCY were 0.522 and 0.069 mg/L, respectively, which were significantly lower than those of bloodstream isolates, 0.812 and 0.113 mg/L, respectively (*P* < 0.05).

### MIC Values of the Nine Drugs Compared Within *Candida parapsilosis* Complex Isolates

The GM MIC of bloodstream *C. parapsilosis* for FLZ (0.704 mg/L) was significantly lower than that of *C. metapsilosis* (0.997 mg/L) (*P* < 0.05, Figure 1 and Supplementary Table 1). The GM MIC of bloodstream *C. parapsilosis* for MFG (0.745 mg/L) was significantly higher than those of *C. metapsilosis* (0.264 mg/L) and *C. orthopsilosis* (0.518 mg/L) (*P* < 0.05). The GM MIC of bloodstream *C. parapsilosis* for VRZ (0.015 mg/L) was significantly lower than those of *C. metapsilosis* (0.023 mg/L) and *C. orthopsilosis* (0.041 mg/L) (*P* < 0.05). The GM MIC of bloodstream *C. orthopsilosis* for ITZ (0.113 mg/L) was significantly higher than that of *C. parapsilosis* (0.061 mg/L) (*P* < 0.05). The GM MIC of bloodstream *C. orthopsilosis* for POZ (0.085 mg/L) was significantly higher than those of *C. parapsilosis* (0.033 mg/L) and *C. metapsilosis* (0.032 mg/L) (*P* < 0.05). The GM MICs of bloodstream *C. metapsilosis* for CAS (0.115 mg/L), AFG (0.150 mg/L), and FCY (0.051 mg/L) were significantly lower than those of *C. parapsilosis* (0.464, 0.918, and 0.136 mg/L, respectively) and *C. orthopsilosis* (0.378, 0.812, and 0.113 mg/L, respectively) (*P* < 0.05).

### MIC Values of Bloodstream *Candida parapsilosis* Isolates With the Nine Drugs Compared Over Time

**FIGURE 2:**
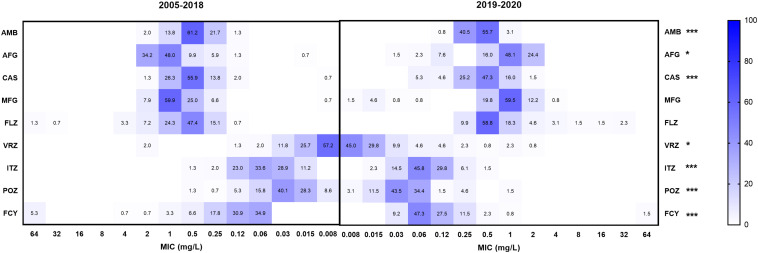
Comparison of the proportions of minimum inhibitory concentration (MIC) distributions of bloodstream *C. parapsilosi*s isolates between 2005–2018 and 2019–2020. *Indicates 0.01 < *P* < 0.5, statistically significant differences of MIC values between 2005–2018 and 2019–2020; **indicates 0.001 < *P* < 0.01, ***indicates *P* < 0.001. FLZ, fluconazole; VRZ, voriconazole; ITZ, itraconazole; POZ, posaconazole; MFG, micafungin; AFG, anidulafungin; CAS, caspofungin; AMB, amphotericin B; FCY, 5-flucytosine.

The MIC distributions of *C. parapsilosis* isolates with the nine drugs were different between 2005–2018 (152 strains) and 2019–2020 (131 strains) (Figure 2). The GM MICs of strains collected from 2019 to 2020 for VRZ, POZ, and ITZ were 0.018, 0.040, and 0.073 mg/L, respectively, which were significantly higher than those from 2005 to 2018 (0.013, 0.028, and 0.052 mg/L, respectively) (*P* < 0.05). In contrast, GM MICs of the strains collected from 2019 to 2020 for AMB, AFG, CAS, and FCY were 0.382, 0.801, 0.401, and 0.096 mg/L, respectively, which were significantly lower than those from 2005 to 2018 (0.430, 0.918, 0.464, and 0.136 mg/L, respectively) (*P* < 0.05).

### MIC Results for ISA

A total of 143 *C. parapsilosis* complex isolates were tested for antifungal susceptibility to ISA (Table 3). The isolates all showed great susceptibility to the drug, with MIC ranges ≤ 0.008–1 mg/L.

**TABLE 3:** *In vitro* susceptibilities to isaconazole of the 143 *C. parapsilosis* complex isolates.

MIC terms (mg/L)	*C. parapsilosis*		*C. metapsilosis*		*C. orthopsilosis*	
			
	Blood (*n* = 61)	Non-blood (*n* = 56)	Blood (*n* = 10)	Non-blood (*n* = 12)	Blood (*n* = 2)	Non-blood (*n* = 2)
MIC range	≤0.008–1	≤0.008–0.12	≤0.008–0.03	≤0.008–0.03	≤0.008–0.06	0.015–0.12
MIC50	≤0.008	0.015	≤0.008	≤0.008		
MIC90	0.015	0.03	0.03	0.015		
Modal MIC	≤0.008	0.015	≤0.008	≤0.008	≤0.008, 0.06	0.015, 0.12
GM MIC	0.012	0.012	0.012	0.010	0.022	0.042

*MIC, minimum inhibitory concentration; MIC50, the MICs required to inhibit the growth of 50% of the organisms; MIC90, the MICs required to inhibit the growth of 90% of the organisms; GM, geometric mean value.*

## Discussion

*Candida parapsilosis* has become the second most prevalent non-*albicans Candida* species that causes invasive infections worldwide, ranging from 24.3% in Latin America to 12.9% in Asia-Pacific. From 2010 to 2019 in Beijing, the most frequently identified non-*albicans Candida* species were *C. parapsilosis*, *C. tropicalis*, and *C. glabrata* (Song et al., 2020). *C. orthopsilosis* and *C. metapsilosis* were reported to have much lower proportions (<1% of all *Candida* species) (Pfaller et al., 2019; Castanheira et al., 2020). Among the *C. parapsilosis* complexes that cause candidemia, *C. orthopsilosis* is responsible for approximately 40% in other countries, following *C. parapsilosis* with about 60%, both of which are much higher than the results in this study (Maria et al., 2018; Vigezzi et al., 2019). The distribution of *C. parapsilosis* (89.6%), *C. metapsilosis* (4.1%), and *C. orthopsilosis* (6.3%) from bloodstream (Table 2) is similar to what has been reported in Iran and Venezuela (Moreno et al., 2017; Arastehfar et al., 2019). Furthermore, this study also investigated the non-bloodstream *C. parapsilosis* complex, and there were nearly 1.8 times more of these isolates than bloodstream ones (Supplementary Table 1). The non-bloodstream sources included sputum, urine, stool, CVC, secretions, and the ear, by rank. Our data are consistent with the theory that *C. parapsilosis* complex species are a group of opportunistic invasive pathogenic fungi that often colonize the skin, mucous membranes, respiratory tract, intestinal tract, and reproductive tract, causing infections when the host’s immunity is impaired. The species can also form biofilms on CVC and other medically implanted devices. The possibility of nosocomial cross-infections *via* the hands of the medical staff is alarming (Trofa et al., 2008; van Asbeck et al., 2009; Wang et al., 2016; Escribano et al., 2018; Tóth et al., 2019; Zhang et al., 2020).

The increasing number of nosocomial *C. parapsilosis* complex infections has raised concerns about conducting antifungal susceptibility tests to optimize clinical treatments (van Asbeck et al., 2008; Gonçalves et al., 2010; Cantón et al., 2011; Pemán et al., 2012; Ruiz et al., 2013; Gago et al., 2014). CLSI M60-Ed2 updated the application rules for clinical breakpoints of *C. parapsilosis* complex: if a cryptic species of the complex is identified, the ECVs should be used instead of the breakpoints (CLSI, 2020). Therefore, this study categorized *C. metapsilosis* and *C. orthopsilosis* as WT and NWT based on the ECVs. Overall, the susceptible rates of bloodstream *C. parapsilosis* to AFG, CAS, MFG, FLZ, and VRZ ranged between 93.3 and 100%, its WT rates to ITZ, POZ, and AMB were between 98.6 and 99.6%, similar to the findings of other studies (da Silva et al., 2015; Moreno et al., 2017; Pfaller et al., 2019). The WT rates for bloodstream *C. metapsilosis* and *C. orthopsilosis* were between 92.3 and 100%, while there were 15% NWT for *C. orthopsilosis* for both FLZ and VRZ (Supplementary Table 2). Similarly, the data by the Shanghai Invasive Fungi Infection Group (IFIG) showed that the resistance rate of *C. parapsilosis* increased from 0 to approximately 5% between 2017 and 2019 (data not published).

In this study, we also investigated the antifungal susceptibilities of non-bloodstream isolates. Notably, the MICs of non-bloodstream *C. parapsilosis* to CAS, MFG, FLZ, VRZ, ITZ, and POZ were significantly higher than those of bloodstream isolates (*P* < 0.05). This was also true for the MICs of *C. metapsilosis* to FCY (*P* < 0.05). However, MICs of non-bloodstream *C. orthopsilosis* to AFG and FCY were significantly lower than those of bloodstream isolates (*P* < 0.05, Figure 1). Interestingly, the resistance rate of *Klebsiella pneumoniae* isolated from sputum also tends to be higher than those isolated from blood (Zhao-Yun et al., 2017; Feng et al., 2020). Because *C. parapsilosis* complex can transform from colonizing to invasive phenotypes (Escribano et al., 2018), the antifungal treatment and management of non-bloodstream species should be subject to strict supervision as for bloodstream species (Zhang et al., 2015; Pappas et al., 2018).

The supervision and management of antifungal treatments was performed by ECIFIG, which was launched in 2019 in Shanghai. Since launching, ECIFIG has been identifying organisms, testing their antifungal susceptibilities, and advocating the correct and rational use of antifungal drugs. It is believed that inappropriate drug exposure drives resistance. The mechanisms that cause drug resistance may naturally occur in less susceptible species and are then acquired in strains of susceptible organisms. Compared with MICs from 2005 to 2018, MIC values of bloodstream *C. parapsilosis* isolates collected from 2019 to 2020 decreased significantly for AMB, AFG, CAS, and FCY but increased significantly for VRZ, POZ, and ITZ (*P* < 0.05, Figure 2). This change indicated that attention should be paid to the clinical and agricultural use of azoles in Eastern China. It is also reflected in the opinion that an effective antifungal stewardship program is significant for controlling drug resistance.

The ECVs published in CLSI M59 are different among *C. parapsilosis* complex species for AMB, AFG, CAS, FLZ, ITZ, MFG, and VRZ. In this study, similar to the findings in CLSI M59, the MIC values of bloodstream *C. metapsilosis* were significantly lower than those of *C. parapsilosis* and *C. orthopsilosis* for AFG and CAS. MICs of *C. parapsilosis* were significantly higher than those of *C. metapsilosis* and *C. orthopsilosis* for MFG (Figure 1). However, MIC values of *C. parapsilosis* were significantly higher than those of *C. metapsilosis* for FLZ, while the ECV of *C. parapsilosis* for FLZ was lower than that of *C. metapsilosis*. Additionally, MICs of *C. orthopsilosis* were significantly higher than those of *C. parapsilosis* for ITZ and were also significantly higher than those of *C. parapsilosis* and *C. metapsilosis* for POZ. The ECVs for ITZ were equal for *C. parapsilosis* and *C. orthopsilosis*, and the ECVs for POZ were the same for all three species. This indicated that the MIC distributions of *C. parapsilosis* for FLZ and the distributions of *C. orthopsilosis* for ITZ and POZ might be higher than those in the CLSI SENTRY program (Pfaller et al., 2011a,b, 2012, 2019; Pfaller, 2012; Castanheira et al., 2016). FLZ is recommended as first-line therapy for candidemia (Pappas et al., 2016), and clonal transmission of FLZ-resistant strains has been reported in Brazil and India (Govender et al., 2016; Thomaz et al., 2018; Singh et al., 2019). Therefore, the higher MIC distributions for azoles in this study is concerning. Apart from that, the MIC values for ISA in this study seemed quite low for *C. parapsilosis* complex isolates. This drug has shown potential as an alternative for candidemia treatment.

## Conclusion

In summary, the higher MIC values of non-bloodstream isolates compared with bloodstream isolates should arouse attention for antifungal treatment and management of non-bloodstream isolates. Additionally, the increased MIC values for azoles of bloodstream *C. parapsilosis* isolates over the years and the higher MIC distributions for azoles in this study than in CLSI M59 raise concerns about the proper use of azoles in the clinic and environment. It may be worth establishing Eastern China’s own ECV for *C. parapsilosis* complex to incorporate better clinical treatment and therapeutic drug monitoring. Finally, ISA has the potential to be an alternative treatment for candidemia.

## Data Availability Statement

The original contributions presented in the study are included in the article/Supplementary Material, further inquiries can be directed to the corresponding author/s.

## Ethics Statement

All strains isolated from patients were preserved in the ECIFIG at Shanghai East Hospital. We conducted a retrospective study on the isolates and patient data, including age and gender from electronic laboratory records. The need for informed consent was waived by the Clinical Research Ethics Committee.

## Author Contributions

WW, LZ, and HX designed the experiments and supervised data analysis. JG, HS, DQ, and LW performed the antifungal susceptibility testing. JG and MZ wrote the manuscript. HX, LL, YL, CW, HD, HL, WZ, YWe, HZ, WX, YZ, SZ, RT, DW, and YWa collected and analyzed the data. All authors discussed the results and commented on the manuscript.

## Conflict of Interest

The authors declare that the research was conducted in the absence of any commercial or financial relationships that could be construed as a potential conflict of interest.
