# Supplementary material for: Prevalence and Antifungal Susceptibility of Candida parapsilosis Species Complex in Eastern China: A 15-Year Retrospective Study by ECIFIG
Source: Front Microbiol. 2021 Mar 4;12:644000. doi: 10.3389/fmicb.2021.644000 (PMC7969513; doi:10.3389/fmicb.2021.644000)
Supplement: Supplementary file 1 [file Data_Sheet_1.docx]

Table S1. *In vitro* susceptibilities of nine antifungal drugs for bloodstream and non-bloodstream *C. parapsilosis* complex isolates.

| **Sources** | **Species** | **MIC terms** | **MIC values (mg/L)** | | | | | | | | |
| --- | --- | --- | --- | --- | --- | --- | --- | --- | --- | --- | --- |
|  |  |  | **AMB** | **AFG** | **CAS** | **FLZ** | **FCY** | **ITZ** | **MFG** | **POZ** | **VRZ** |
| blood | *C. parapsilosis* (n=283) | MIC range | ≤0.12-2 | ≤0.015-2 | ≤0.008-2 | ≤0.12-64 | ≤0.03-64 | ≤0.015-0.5 | ≤0.008-4 | ≤0.008-1 | ≤0.008-2 |
|  |  | MIC50 | 0.5 | 1 | 0.5 | 0.5 | 0.12 | 0.06 | 1 | 0.03 | ≤0.008 |
|  |  | MIC90 | 0.5 | 2 | 1 | 2 | 0.5 | 0.12 | 2 | 0.06 | 0.03 |
|  |  | Modal MIC | 0.5 | 1 | 0.5 | 0.5 | 0.06 | 0.06 | 1 | 0.03 | ≤0.008 |
|  |  | GM MIC | 0.430 | 0.918 | 0.464 | 0.704 | 0.136 | 0.061 | 0.745 | 0.033 | 0.015 |
|  | *C. metapsilosis* (n=13) | MIC range | ≤0.12-0.5 | ≤0.015-1 | 0.06-0.5 | ≤0.12-2 | ≤0.03-2 | ≤0.015-0.12 | ≤0.008-0.5 | ≤0.008-0.12 | ≤0.008-0.06 |
|  |  | MIC50 | 0.5 | 0.12 | 0.12 | 1 | ≤0.03 | 0.06 | 0.25 | 0.03 | 0.03 |
|  |  | MIC90 | 0.5 | 0.5 | 0.25 | 2 | 0.25 | 0.12 | 0.5 | 0.06 | 0.03 |
|  |  | Modal MIC | 0.5 | 0.12 | 0.06, 0.12 | 1, 2 | ≤0.03 | 0.06, 0.12 | 0.25, 0.5 | 0.03, 0.06 | 0.03 |
|  |  | GM MIC | 0.362 | 0.150 | 0.115 | 0.997 | 0.051 | 0.063 | 0.264 | 0.032 | 0.023 |
|  | *C. orthopsilosis* (n=20) | MIC range | 0.25-1 | 0.25-2 | 0.12-2 | 0.25-64 | ≤0.03-2 | 0.03-0.5 | 0.25-2 | 0.03-0.5 | ≤0.008-2 |
|  |  | MIC50 | 0.5 | 1 | 0.25 | 0.5 | 0.12 | 0.12 | 0.5 | 0.06 | 0.03 |
|  |  | MIC90 | 1 | 1 | 0.5 | 16 | 0.25 | 0.25 | 0.5 | 0.12 | 0.25 |
|  |  | Modal MIC | 0.5 | 1 | 0.25 | 0.5 | 0.12 | 0.12 | 0.5 | 0.12 | 0.03 |
|  |  | GM MIC | 0.467 | 0.812 | 0.378 | 1.072 | 0.113 | 0.113 | 0.518 | 0.085 | 0.041 |
| non-blood | *C. parapsilosis* (n=480) | MIC range | ≤0.12-2 | 0.03-4 | 0.015-8 | 0.25-128 | ≤0.03-64 | ≤0.015-2 | ≤0.008-2 | ≤0.008-1 | ≤0.008-2 |
|  |  | MIC50 | 0.5 | 1 | 0.5 | 0.5 | 0.12 | 0.06 | 1 | 0.06 | 0.015 |
|  |  | MIC90 | 1 | 2 | 1 | 2 | 0.25 | 0.12 | 2 | 0.12 | 0.06 |
|  |  | Modal MIC | 0.5 | 1 | 0.5 | 0.5 | 0.06 | 0.12 | 1 | 0.06 | 0.015 |
|  |  | GM MIC | 0.450 | 0.949 | 0.555 | 0.816 | 0.123 | 0.076 | 0.853 | 0.042 | 0.017 |
|  | *C. metapsilosis* (n=59) | MIC range | ≤0.12-2 | 0.06-2 | 0.03-1 | 0.5-4 | ≤0.03-4 | ≤0.015-0.25 | 0.25-1 | 0.015-0.25 | 0.015-0.12 |
|  |  | MIC50 | 0.25 | 0.12 | 0.25 | 2 | 0.06 | 0.12 | 0.5 | 0.03 | 0.03 |
|  |  | MIC90 | 1 | 1 | 0.5 | 2 | 0.12 | 0.25 | 0.5 | 0.12 | 0.06 |
|  |  | Modal MIC | 0.25 | 0.12 | 0.25 | 2 | 0.06 | 0.12 | 0.25 | 0.03 | 0.03 |
|  |  | GM MIC | 0.429 | 0.223 | 0.181 | 1.491 | 0.071 | 0.088 | 0.364 | 0.043 | 0.026 |
|  | *C. orthopsilosis* (n=29) | MIC range | 0.12-1 | 0.12-2 | 0.12-2 | 0.25-64 | 0.06-0.25 | 0.03-0.5 | 0.12-4 | 0.015-0.5 | 0.008-2 |
|  |  | MIC50 | 0.5 | 0.5 | 0.5 | 1 | 0.06 | 0.12 | 0.5 | 0.06 | 0.03 |
|  |  | MIC90 | 1 | 1 | 1 | 4 | 0.12 | 0.25 | 0.5 | 0.12 | 0.12 |
|  |  | Modal MIC | 0.5 | 1 | 0.5 | 1 | 0.06 | 0.12 | 0.5 | 0.06 | 0.03 |
|  |  | GM MIC | 0.356 | 0.522 | 0.401 | 1.182 | 0.069 | 0.104 | 0.476 | 0.068 | 0.039 |

*MIC, Minimum inhibitory concentration; MIC50, the MICs required to inhibit the growth of 50% of the organisms; MIC90, the MICs required to inhibit the growth of 90% of the organisms; GM, Geometric mean value;* *FLZ, Fluconazole; VRZ, Voriconazole; ITZ, Itraconazole; POZ, Posaconazole; MFG, Micafungin; AFG, Anidulafungin; CAS, Caspofungin; AMB, Amphotericin B; FCY, 5-Flucytosine.*

Table S2. Susceptibility categories of eight antifungal drugs for bloodstream and non-bloodstream *C. parapsilosis* complex isolates.

| **Sources** | **Species** | **Categories** | **No. of isolates (%)** | | | | | | | |
| --- | --- | --- | --- | --- | --- | --- | --- | --- | --- | --- |
|  |  |  | **AFG** | **CAS** | **MFG** | **FLZ** | **VRZ** | **ITZ** | **POZ** | **AMB** |
| blood | *C.parapsilosis* | S | 283(100) | 283(100) | 282(99.6) | 264(93.3) | 272(96.1) |  |  |  |
|  |  | I |  |  | 1(0.4) |  | 4(1.4) |  |  |  |
|  |  | SDD |  |  |  | 9(3.2) |  |  |  |  |
|  |  | R |  |  |  | 10(3.5) | 7(2.5) |  |  |  |
|  |  | NWT |  |  |  |  |  | 1(0.4) | 4(1.4) | 3(1.1) |
|  |  | WT |  |  |  |  |  | 282(99.6) | 279(98.6) | 280(98.9) |
|  | *C.metapsilosis* | NWT | 1(7.7) | 1(7.7) |  |  |  |  |  |  |
|  |  | WT | 12(92.3) | 12(92.3) | 13(100) | 13(100) | 13(100) | 13(100) | 13(100) | 13(100) |
|  | *C.orthopsilosis* | NWT |  | 1(5) | 1(5) | 3(15) | 3(15) |  | 1(5) |  |
|  |  | WT | 20(100) | 19(95) | 19(95) | 17(85) | 17(85) | 20(100) | 19(95) | 20(100) |
| non-blood | *C.parapsilosis* | S | 473(98.5) | 477(99.4) | 480(100) | 444(92.5) | 467(97.3) |  |  |  |
|  |  | I | 7(1.5) | 2(0.4) |  |  | 6(1.3) |  |  |  |
|  |  | SDD |  |  |  | 21(4.4) |  |  |  |  |
|  |  | R |  | 1(0.2) |  | 15(3.1) | 7(1.5) |  |  |  |
|  |  | NWT |  |  |  |  |  | 1(0.2) | 3(0.6) | 35(7.3) |
|  |  | WT |  |  |  |  |  | 479(99.8) | 477(99.4) | 445(92.7) |
|  | *C.metapsilosis* | NWT | 6(10.2) | 12(20.3) |  |  | 2(3.4) |  |  | 1(1.7) |
|  |  | WT | 53(89.8) | 47(79.7) | 59(100) | 59(100) | 57(96.6) | 59(100) | 59(100) | 58(98.3) |
|  | *C.orthopsilosis* | NWT |  | 1(3.4) | 1(3.4) | 3(10.3) | 2(6.9) |  | 1(3.4) |  |
|  |  | WT | 29(100) | 28(96.6) | 28(96.6) | 26(89.7) | 27(93.1) | 29(100) | 28(96.6) | 29(100) |

*FLZ, Fluconazole; VRZ, Voriconazole; ITZ, Itraconazole; POZ, Posaconazole; MFG, Micafungin; AFG, Anidulafungin; CAS, Caspofungin; AMB, Amphotericin B; FCY, 5-Flucytosine; S, Susceptible; I, Intermediate; SDD, Susceptible-dose dependent; R, Resistant; WT, Wild type; NWT, None-wild type.*
